# Predisposition to Change Is Linked to Job Satisfaction: Assessing the Mediation Roles of Workplace Relation Civility and Insight

**DOI:** 10.3390/ijerph17062141

**Published:** 2020-03-23

**Authors:** Alessio Gori, Eleonora Topino

**Affiliations:** 1 Department of Health Sciences, University of Florence, via di San Salvi 12, pad. 26, 50135 Firenze, Italy; 2 Department of Human Sciences, LUMSA University of Rome, Via della Traspontina 21, 00193 Rome, Italy; eleonora.topino@gmail.com

**Keywords:** predisposition to change, workplace relational civility, insight, job satisfaction

## Abstract

The globalization processes typical of liquid modern society require organizations to have high levels of flexibility, dynamism, and rapidity of change, testing the adaptability of workers with possible repercussions on well-being and productivity. Therefore, this study aimed to investigate the role of several psychological factors in favoring job satisfaction in a group of organizational workers (mean age = 46.24; SD = 9.99; 40.9% males and 59.1% females). Firstly, the impact of predisposition to change on job satisfaction through workplace relational civility (others with me) or insight orientation as independent mediating variables was analyzed. After that, this relationship was also studied by testing the effect that the simultaneous interaction of both mediators could have. Results show that workplace relational civility (others with me) significantly mediated the relationship between predisposition to change and job satisfaction, while no significance was found in the effect of insight when considered individually. However, the latter acquires greater relevance if placed in interaction with the other mediator, that is found to be the most proximal factor linking job satisfaction to the other more distal variables. Such findings might have a relevant role in strengthening preventive intervening, favoring positive results for greater well-being of both subjects and organizations.

## 1. Introduction

Rethinking work and work contexts promoting health is crucial in the liquid environments of the XXI century [1,2,3]. The hours spent working represent a very significant part of people’s daily lives; it is therefore understandable how the sense of satisfaction for one’s job is strictly connected to individual well-being [4,5,6,7] as well as to one’s productivity at work [8,9,10,11]. Specifically, several studies found that job dissatisfaction correlates not only with absenteeism, turnover, poor job performance, poor empathy with customers [12,13,14,15] but also with psychological distress and disorders such as burnout, depression, anxiety, and low self-esteem [16,17,18]. Over the years, many definitions of job satisfaction have followed one another in in the Literature (see, for a review Reference [19]), but almost all agree in considering it as an attitude. Indeed, the most common one remains that of Locke [20] which integrates emotional, cognitive, and behavioral aspects:

“A pleasurable or positive emotional state resulting from the appraisal of one’s job as attaining or allowing the attainment of one’s important job values, providing these values are congruent with or help to fulfill one’s basic needs. These needs are of two separable but interdependent types: bodily or physical needs and psychological needs”.

[20] (p. 1319)

Resuming some concepts of Maslow’s theory [21], work can therefore be seen as a tool to satisfy the individuals’ primary needs (allowing survival and safety), but potentially it is also a means to gratify the desires for affection, esteem, and self-affirmation: in other words, together with the salary which allows to meeting basic needs, workers also seek other job characteristics that can contribute to the status, recognition, sense of freedom or even to the self-assertion and maximization of one’s own potential [22]. This therefore highlights the complexity underlying the concept of job satisfaction, which can be influenced by the interaction of both personal and environmental characteristics [23] that also tend to have different effects based on gender (e.g., men tend to give great importance to the relationship aspect, while women are influenced a lot by “working conditions”) [24]. Moreover, further nuances are added in today’s world which, driven by profound and rapid technological innovations, requires workers to have high levels of flexibility, specialization, and dedication but also the ability to constantly renew and update themselves to sustain the strong impact that globalization is having on the world of employment in postmodern society [25]. According to this framework, therefore, the aim of this study was to investigate the role of several variables in contributing to job satisfaction. Specially, particular attention was paid to the linkage between predisposition to change and job satisfaction, also examining the role of insight orientation and workplace relational civility (others with me) in mediating this association.

The mutable market conditions push organizations towards the implementation of rapid internal changes in structure, objectives, technology or activity [26] in order to effectively adapt to the iridescent outside world: this, of course, has repercussions on workers, whom are asked to accommodate these rapid changes [27]. However, tackling change can be very difficult [28,29] and the resulting uncertainty can be a source of resistance and tendency to maintain the status quo [30,31,32]. This, in addition, hinders the development of effective change strategies [33,34,35], has a negative impact on professional satisfaction [36,37,38], influencing the intention of turnover [38,39,40]. On the other hand, change can also be approached with acceptance, seeing in it an opportunity to grow [41,42,43]: the workers who manage to maintain this openness usually appear more resilient, demonstrating higher levels of self-esteem and optimism [28]. Specifically, predisposition to change is the ability to learn from it, considering it as a means of improving one’s quality of life [44]. Several studies show how this attitude of acceptance is a key factor to mobilize and face the job challenges that current markets impose, influencing not only the ability to tolerate organizational change but also leading towards a general state of well-being [45,46]. In this way, a better contextual adaptation is facilitated, which by definition implies the ability to cope with the dynamic and reciprocal influence of all contextual biopsychosocial dimensions, allowing for a more effective management of all the environmental stimuli including the social one [47]. Thus, work continues to be a source of fulfillment for the needs of self-determination and survival, which goes together with those of social connection [48,49]. With reference to this last aspect, it should be emphasized that humans are social creatures, and this is also reflected in the organizational context [50]: a lack of coworker support, in fact, can induce employees to feel isolated aggravating distress [51], while perceiving the assistance of a good social network within the job environment allows to keep high motivation, self-esteem, and self-confidence, and increasing productivity and rapid management of duties [52,53]. These positive interactions among colleagues provide, in fact, a way to satisfy the need for relationships, which concerns the basic desire for reciprocity, connection, and taking care of others [54]. In line with social exchange theory, the more a worker feels considered, appreciated, and valued by coworkers, the more he will tend to reciprocate by loving his job, developing loyalty to the organization, and committing himself to the pursuit of common objectives: the workplace relational civility perceived by the employee will lead him to see the job environment in a favorable light, making him feel a satisfied member of a mutual civility obligations network [55,56,57]. As a confirmation of this, several studies highlight how high levels of job satisfaction can be maintained even in the presence of difficulties, if a positive, empathetic, compassionate, and supportive attitude of colleagues is perceived [58,59]. However, both in relating to others and in managing difficult situations, a key element seems to be good self-understanding [60,61]. Work activity is a core aspect in determining part of the individuals’ identity [62,63] and, therefore, organizational changes can generate fear and stress [64], reducing the clarity of self-concept and undermining one’s sense of security [65,66]. However, it is also true that the perception of transition phases as sources of new and stimulating challenges can instead lead to a desire of growth, facilitating the acquisition of new perspectives, resources, and identity meanings, reaching a greater insight of previously unrecognized thoughts, feelings or behaviors [67]. Therefore, insight develops, and changes are influenced by circumstances and events just like the working environment itself [68] and can vary from superficial knowledge to a profound elaboration of previously unprocessed content [69]. Several studies have shown that this perception of self-expansion in the workplace is associated with greater commitment and satisfaction [70] thanks to a better understanding of both the self and one’s emotions which appears extremely useful for the management of changing and unpredictable working conditions in the 21st century [71]. Insight is a complex construct, originally finding frequent application in the psychotherapeutic field but which can also be useful in the world of work. Over time, various definitions have been developed with common denominators in the concepts of “understanding” and “connection” between past and present experiences as well as between thoughts, feelings, desires, and behaviors [72]. Furthermore, several authors increase its complexity by enriching it with relational aspects: the insight does not only concern self-awareness, but also that of the own’s interpersonal models [73], and it is precisely in recognition by others that one can develop a better understanding of the self [74]. Therefore, in the working context, the favorable and supportive attitude towards changes facilitates the acquisition of both healthy organizational maturity and new worker paradigms of meaning [7,16], characterized by a polycentric vision in which well-being is common and shared [75]. In this perspective, the close relationship between workplace relational civility and insight is evident, and they influence each other evolving hand in hand with the changes that the worker must face: in other words, contextual adaptation creates a bridge between the internal, external, and relational worlds [47].

**Figure 1:**
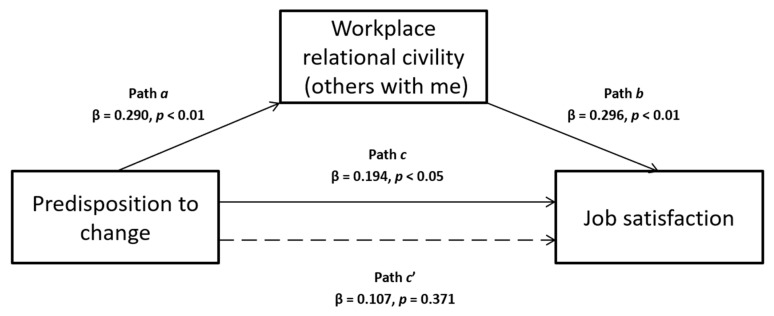
Relationship between predisposition to change and job satisfaction with workplace relational civility (others with me) as mediator.

**Figure 2:**
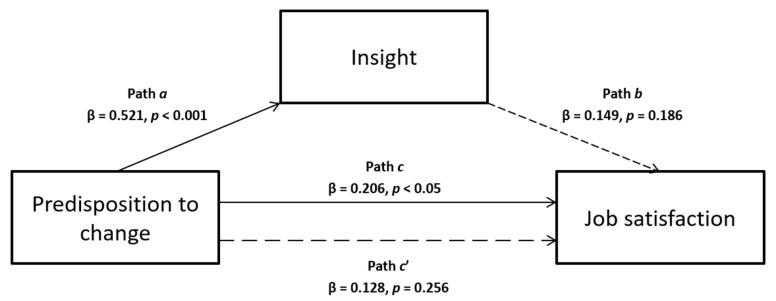
Relationship between predisposition to change and job satisfaction with insight as mediator.

**Figure 3:**
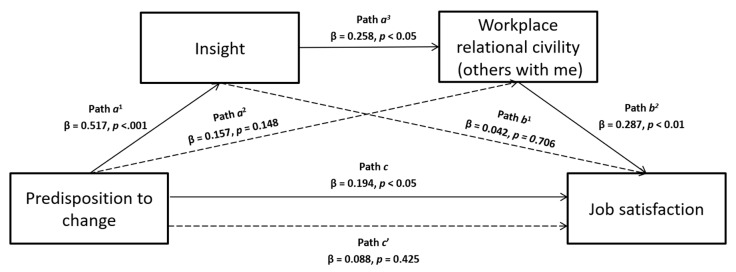
A serial multiple mediation model with predisposition to change as an independent variable, job satisfaction as the outcome, and both insight and workplace relational civility (others with me) as mediators, supported by analyzes.

**Figure 4:**
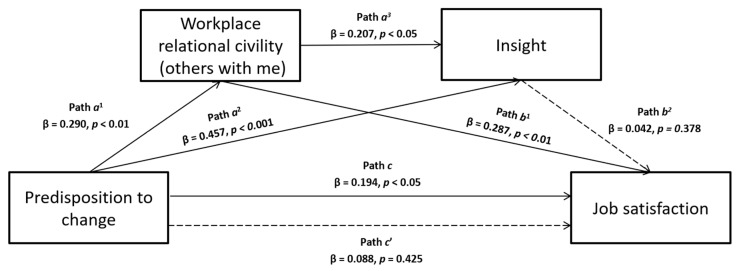
A serial multiple mediation model with predisposition to change as the independent variable, job satisfaction as the outcome, and both workplace relational civility (others with me) and insight as mediators, not supported by analyzes.

Based on the literature previously presented, this study aimed to verify several hypothesized mediation models. In particular, the first two are single mediations, in which we assume that predisposition to change can influence job satisfaction, that predisposition to change predicts workplace relational civility (others with me) or insight orientation (see Figure 1 and Figure 2, respectively), and that the latter two can be associated to job satisfaction. Following, the last two models are multiple mediations, in which we hypothesize that the influence of predisposition to change on job satisfaction is mediated simultaneously by both insight orientation and workplace relational civility (others with me), and we tested their effect presenting them in different order for each model (see Figure 3 and Figure 4).

## 2. Materials and Methods 

### 2.1. Participants and Procedure

The sample was composed of 130 participants (54 men and 78 women) with a mean age of 46.24 years old (SD = 9.99). All of them were randomly recruited and among the workers of some public and private organizations in Tuscany (Italy): 62.1% were canteen staff, 24.2% were public green spaces maintenance employees, and 13.6% were workers in road haulage. In terms of educational status, most of the individuals had a lower or higher secondary school diploma (53.8% and 19.7%, respectively), while 5.3% attended only primary school and 4.5 had a university degree (see Table 1). Participants completed the self-report measures after an explanation of the study’s general purpose and procedures and after giving written informed consent with the knowledge that they were free to leave the research at any time. They did not receive any form of compensation for their participation in the study and their anonymity was guaranteed. The research was conducted in accordance with the ethical standards, consistently with the latest version of the Declaration of Helsinki (ethical approval number 001, 04/09/2017) [76].

### 2.2. Measures

#### 2.2.1. The Acceptance of Change Scale (ACS)

The Acceptance of Chance Scale (ACS) [44] is a self-report scale designed to measure the tendency to accept or move toward change. It consists of five factors (each of 4 items) which were assessed using 20 items on a 5 point Likert scale (from 1 = “not at all” to 5 = “a great deal”). The dimensions were: positive reaction to change (e.g., “I can find the positives in changes that are apparently negative”); change seeking (e.g., “I am looking for changes in my life, even when things are going well”); cognitive flexibility (e.g., “5. If necessary, it is not difficult for me to change my mind”); predisposition to change (e.g., “I easily identify alternative paths”); and support for change (e.g., “I trust the people close to me when faced with change”). Di Fabio and Gori [44] concluded that the ACS has good psychometric properties and both satisfactory confirmatory factor analysis (CFA) goodness-of-fit indices (CFI = 0.94; TLI = 0.92; SRMR = 0.05; RMSEA = 0.05 even if the chi-square was significant at *p* < 0.001) and internal consistency values (Predisposition to Change, α = 0.83; Support for Change, α = 0.79; Change Seeking, α = 0.80; Positive Reaction to Change, α = 0.75; Cognitive Flexibility, α = 0.72, and the total scale α = 0.88). In the present research, only the subscale Predisposition to Change was used which showed a Cronbach’s alpha of 0.87 (although good indices were also found in the total score and in the other subscales in the current sample, with α = 0.91, α = 0.87, α = 0.87, α = 0.84 and α = 0.76, respectively).

#### 2.2.2. Workplace Relational Civility Scale (WRCS)

The Workplace Relational Civility Scale (WRCS) [77] is a self-report measure designed to assess relational civility in the workplace. It consists of 26 items on a 5 point Likert scale (1 = “not at all”, 2 = “a little”, 3 = “somewhat”, 4 = “much”, 5 = “a great deal”) grouped in two mirrored parts (A, focused on self-perception of their general relationship with others over the past 3 months; and B, focused on the individual’s perception of the general relationship of others with him over the same time period), in each of which three dimensions are analyzed: Relational Readiness (RR; 5 item, e.g., “I was attentive to the needs of others” for part A and “Others were attentive to my needs” for part B); Relational Culture (RCu; 4 item, e.g., “I was polite toward others” for part A and “Others were polite toward me” for part B); and Relational Decency (RD, 4 item, e.g., “I respected the opinions of others” for part A and “Others respected my opinions” for part B) at work. The sum of these dimensions allows to have the scores of both part A and B of the WRCS, which, if added together, give the total score. The WRCS is characterized by good psychometric qualities, with satisfactory CFA goodness-of-fit indices (x^2^/df = 1.14 *p* < 0.210, CFI = 0.98, TLI = 0.97, SRMR = 0.03, RMSEA = 0.04 for part A and x^2^/df = 1.93 *p* < 0.001, CFI = 0.93, TLI = 0.92, SRMR = 0.05, RMSEA = 0.10 for part B) and a good internal consistency, with α = 0.87, α = 0.83, α = 0.76 and α = 0.75 (total score, RR, RCu, and Rd, respectively) for Part A and α = 0.92, α = 0.86, α = 0.88, α = 0.85 (total score, RR, RCu, and Rd, respectively) for Part B [77]. These data were also confirmed in the present sample, where only part B (with α = 0.95, α = 0.96, α = 0.86, α = 0.83 for total score, RR, RCu, and Rd, respectively) was used (although part A also had good indices, with Cronbach’s alphas of 0.89, α = 0.99 α = 0.79, α = 0.3 for total score, RR, RCu, and Rd, respectively).

#### 2.2.3. Insight Orientation Scale (IOS)

The Insight Orientation Scale (IOS) [69] is a self-report scale which assess the insight capacity (also including behaviors, feelings and opinions about this construct). It consists of 7 items on a 5 point Likert scale (from 1 = “not at all”, to 5 = “a great deal”) which investigate seven aspects that can be considered central to the insight process: level of consciousness, problem solving, restructuring (behavior change), awareness, complexity (abstraction, depth), surprise, and self-reflectiveness (thoughtfulness). The total score can be calculated by summing the item scores. The scale showed a satisfactory CFA goodness-of-fit indices (NNFI = 0.92; CFI = 0.92; SRMR = 0.04; RMSEA = 0.09 even if the chi-square was significant at *p* < 0.001) and a good level of internal consistency, with a Cronbach’s alpha of 0.77 in the measure development study [69] and of 0.76 in the present sample.

#### 2.2.4. Job Satisfaction Rating Scale

A Job Satisfaction Rating Scale was used to assess self-reported job satisfaction. Participants were asked to indicate their general job satisfaction level on a Likert scale from 1 = “absolutely dissatisfied” to 10 = “absolutely satisfied”.

### 2.3. Statistical Analyses

Collected data were analyzed with the SPSS software (IBM-SPSS 25.0 version, IBM, Armonk, NY, USA) for Windows. Descriptive analyses for both sample characteristics and variables were calculated. Pearson’s *r* correlations were performed to measure the relationships among the constructs. Moreover, a one-way analysis of variance (ANOVA) was carried out to assess the gender differences in predisposition to change, workplace relational civility (part b), insight orientation, and job satisfaction. Then, the SPSS macro program PROCESS [78] was used to verify the mediation models. Firstly, two single mediations (model 4) were performed to assess the mediation role of workplace relational civility (others with me) or insight orientation in the causal relationship between predisposition to change and job satisfaction. After that, two multiple mediations (model 6) were analyzed by entering both mediators simultaneously into the models, in different order for each time. The indirect effect was tested employing both the Sobel test (for single mediation models) and Bootstrapping procedures with 95% of confidence interval at 5000 samples. 

## 3. Results

Descriptive statistics are shown in Table 1 for the sample characteristics and in Table 2 for the variables. Table 2 also reports the Pearson’s *r* correlations between the scales and subscales used in the study.

Results of the one-way ANOVA revealed significant differences for gender on levels of predisposition to change (F_1,126_ = 6.57, *p* < 0.05) and workplace relational civility part B (F_1,127_ = 7.73, *p* < 0.01). For both of these variables, the mean scores for males (M = 13.11, SD = 4.01, and M = 47.11, SD = 10.83, respectively) were significantly higher than the mean scores for females (M = 11.40, SD = 3.51 and M = 41.76, SD = 10.75, respectively). Instead, concerning insight orientation and job satisfaction, there were no substantial gender differences in their mean scores (*p* = 0.291 and *p* = 0.286). Regarding the two single mediation analyses, data showed that the relationship between predisposition to change and job satisfaction was mediated by workplace relational civility part B but not by insight orientation. In particular, the total non-mediated effect (path *c* in Figure 1) of predisposition to change on job satisfaction was significant (β = 0.194, *p* < 0.05). Then, predisposition to change was related to workplace relational civility part B (β = 0.290, *p* < 0.01), the mediator variable (estimating the path *a* in Figure 1), which in turn affected job satisfaction (β = 0.296, *p* < 0.01), estimating the path *b* in Figure 1. So, the effect of predisposition to change on job satisfaction was reduced after controlling workplace relational civility part B (path *c’* in the Figure 1), becoming insignificant (β = 0.107, *p* = 0.371): these results therefore indicated a complete mediation model, with *R*^2^ = 0.118, F(2, 102) = 6.792, *p <* 0.01. Indeed, the indirect effect was significant with both bootstrapping procedure (Boot LLCI = 0.005–Boot ULCI = 0.106) and Sobel test (z = 2.11; *p* < 0.05).

Concerning the second single mediation model, neither the indirect nor the direct effects (path *c’* in Figure 2; β = 0.128, *p* = 0.256) were significant. Specifically, predisposition to change was a significant predictor of insight orientation (path *a* in Figure 2; β = 0.521, *p* < 0.001), but the latter was not related to job satisfaction (path *b* in Figure 2; β = 0.149, *p* = 0.186). This was confirmed by both bootstrapping procedure (Boot LLCI = –0.031 –Boot ULCI = 0.117) and Sobel test (z = 1.29, *p* = 0.20) which identified an insignificant indirect effect.

If workplace relational civility part B and insight orientation were both considered mediators between predisposition to change and job satisfaction, the final effect changed according to the order in which they entered the model. Indeed, the results showed a significant chained mediation model when predisposition to change predicted insight (path *a*^1^ in Figure 3; β = 0.517, *p* < 0.001) which affected workplace relational civility part B (path *a*^2^ in Figure 3; β = 0.258, *p* < 0.05), that in turn influenced job satisfaction (path *b*^2^ in Figure 3; β = 0.287, *p* < 0.01). However, the direct effect of insight orientation to job satisfaction (β = 0.042, *p* = 0.706; path *b*^1^ in Figure 3) was not significant, like those of predisposition to change on both workplace relational civility part B (path *a*^2^ in Figure 3) and Job satisfaction (path *c’* in Figure 3), suggesting a complete mediation after workplace relational civility part B and insight orientation have been controlled (*R*^2^ = 0.119, F_3.101_ = 4.537, *p <* 0.01). The bootstrapping procedure confirmed the statistical stability of this chained mediation model and the significance of this indirect effect (Boot LLCI = 0.001–Boot ULCI = 0.049).

By reversing the mediators’ entry order in the multiple mediation model concerning the relationship between predisposition to change and job satisfaction, the results showed that predisposition to change was a significant predictor of workplace relational civility part B (path *a*^1^ in Figure 4; β = 0.290, *p* < 0.01), which in turn affected insight orientation (path *a*^3^ in Figure 4; β = 0.207, *p* < 0.05), but the latter did not influence job satisfaction (path *b*^2^ in Figure 4; β = 0.042, *p* = 0.378). So, neither the direct effect (path *c’* in Figure 4; β = 0.088, *p* = 0.425) nor the indirect one were significant in this latter multiple mediation model as confirmed by the bootstrapping procedure (Boot LLCI = −0.010 – Boot ULCI = 0.012).

In Table 3, all the models’ effects indices were summarized.

## 4. Discussion

In recent decades, working life has undergone profound changes caused by an increasingly globalized and flexible system. Manifestations of this can be found, for example, in the loosening of the restrictions governing the use of temporary employment contracts [79] or in a profound technologization which facilitates progressive job intrusion into personal life [80]. In other words, even the working context reflects that “liquidity” [1] peculiar of today’s society, characterized by polymorphic and iridescent references and devoid of absolute certainties. This climate of instability and rapidity of change can arouse discouragement, stress, feelings of precariousness, and job insecurity, which inevitably produce psychological suffering [81,82,83]. It is therefore understandable how keeping themselves motivated to work despite adverse conditions, managing the sense of uncertainty by showing flexibility and a good disposition towards change could be seen as fundamental requirements to survive in modern liquid society and finding one’s own realization [84], as well as promoting both organizational functioning and productivity [75,85].

In line with this framework, the present research was conducted with the aim to investigate the role of several psychological factors in favoring job satisfaction in a group of organizational workers. at first, the impact of predisposition to change on job satisfaction through workplace relational civility (others with me) or insight orientation as independent mediating variables was analyzed. After that, this relationship was also studied by testing the effect that the simultaneous interaction of both mediators could have had. 

Firstly, significant gender differences were found on measures of predisposition to change and workplace relational civility (others with me) with higher scores for males. This is in line with previous research, which shows that men were able to derive greater benefits from coworker support [86], while women perceived greater overload when they had to cope with rapid changes [87], appearing extremely sensitive to the stress derived from the introduction of new technologies in the workplace [88]. Considering that innovation implies acceptance of novelty and new behaviors related to it, another important result was the positive and significant association between predisposition to change and job satisfaction. In fact, in line with this, previous studies had already shown a strong link between resistance to change and a sense of dissatisfaction with one’s work [89,90]. On the other hand, openness to change is related to a broad state of satisfaction with one’s life [29] and general well-being [26]. Concerning this latter, several researches highlight how this can be influenced in the organizational context also by the perceived coworkers support [91,92]. Along the same lines, in fact, the results showed that workplace relational civility (others with me) has a significant role as a mediator between predisposition to change and job Satisfaction. A possible interpretation could be that predisposition to change allows to remain positively open to environmental stimuli (including social ones), facilitating the perception of relational support in the work context: this will be a push to achieve one’s goals even by facing the critical periods of organizational transition [29], better managing stress and maintaining a high level of job satisfaction [12,93]. Indeed, the fact that the direct effect in the mediation model (i.e. when the moderator is accounted for) becomes statistically insignificant, highlights that workplace relational civility (others with me) can be considered as a core factor in promoting positive outcomes in terms of job satisfaction. A significant mediation effect with insight orientation was not found individually; however, this acquires greater relevance if placed in interaction with the other mediator. Therefore, the major contribution of this research was the identification of a serial multiple mediation model indicating that predisposition to change was related to job Satisfaction mainly through the influences of insight orientation and workplace relational civility (others with me). In particular, workplace relational civility (others with me) was found to be the most proximal factor that linked job satisfaction to more distal variables of insight orientation and predisposition to change, and this is confirmed by the loss of significance that was revealed after reversing the order of mediators in the model. In line with the Self-Expansion Model [67,94], in the processes of change, workers can reach a further step in the insight of their resources and limits, sometimes also implying openness to the point of view of others, accepting it, and possibly integrating it with one’s own [70]. This, together with a better understanding and management of one’s emotions, will have a positive impact on interpersonal relationships in the workplace [95,96], facilitating the development of positive and mutually supportive links among coworker [97]. Therefore, on the basis of an openness to change and to the organizational challenges that derive from it, individual self-concepts can be partially defined and supported in periods of transition also by the sense of belonging to the group and to the organization [98], leading to greater job satisfaction and commitment [99,100].

This research has several limitations that should be considered. Firstly, the Italian workers involved in the study were not representative of the national population, and this might limit the generalizability of the findings. So, future research could expand the sample by including employees from different geographical areas in Italy or even in other countries, to test the cross-cultural invariance of the results too; in addition, in a larger sample it could be interesting to evaluate the goodness of the models with cross-group comparisons (e.g., based on gender) and invariance analyses. Furthermore, no distinctions were made among the several types of work activity. This could be an important challenge for future research, in the light of a growing literature that highlights how the type of profession or organization can differently impact workers’ well-being, their job satisfaction, and the consequent ability to adapt to changes: see, for example, the high risk of burnout for health care professions [101] or for educational contexts, where several studies have shown the central role of different forms of social and familiar support to combat the phenomenon [102,103,104]. Besides, according to this vision, it may also be useful to consider the world of non-profit organizations, subject to continuous forced changes to follow the constant public sector reform movement and in which innovation could be facilitated by an effective strategic management of the intellectual capital [105]. Another limit of the present research concerns the use of only self-reported measures (which present some of the CFA indicators that do not fit satisfactorily) and the evaluation of job satisfaction with a single-item scale. Future research could use a multimodal approach (for example, adding interview or naturalistic observations) to overcome the issues associated with these kind of tools (e.g., social desirability). Finally, the study was a cross-sectional research design, and this hinders the definitive establishment of causal links among variables of the hypothesized models. Future research should involve a longitudinal study to permit to lead to safer conclusions, capturing the dynamic nature of organizational changes and attitudes related to them over time. 

## 5. Conclusions

This study allowed for a greater understanding of the variables that play a core role in increasing organizational and workers’ health and productivity and to view the complexity of the current working world. Indeed, this research underlines the effect of insight orientation and workplace relational civility (others with me) in mediating the relationship between predisposition to change and job satisfaction. This adds an additional piece to a growing body of research that analyzes the employees’ attitudes and dispositions towards one’s job [106] which in turn will inevitably influence the organizational outcomes [107]. In this perspective, therefore, the present study provides a further contribution to increase the effectiveness of preventive intervening in favor of positive results for greater well-being of subjects and organizations. In primary prevention perspective [81,108] and strength-based prevention perspectives [109,110], insight orientation and workplace relational civility could represent prevention promising resources because they could be promoted with early specific training for healthy workers and healthy organizations [111,112,113,114,115]. Concerning predisposition to change, an application cue can be also provided in the light of several research underlining how employees’ attitude towards organizational change can be "*shaped by experiences in the work context*" [116] (pp. 640–641) and, therefore, may be susceptible to variations leaded by situational experiences. More specifically, the scientific literature highlights the importance of perceived quality of received information [43], of feeling involved in the decision-making process [28,117], of trust in leaders [117,118], and of a positive track record of past changes in the organization [118] in favoring both openness and support from employees towards change.

## Figures and Tables

**Table 1:** Demographic characteristics of the sample (*n* = 130).

Characteristics		M ± SD	*n*	%
	Age	46.24 ± 9.99		
Sex				
	Males		54	40,9
	Females		78	59.1
Education				
	Elementary school (5 years)		7	5.3
	Middle school diploma (8 years)		71	53.8
	High school diploma (13 years)		26	19.7
	University degree (16 years)		6	4.5
	Missing values		22	16.7
Employment				
	Canteen		82	62.1
	Road haulage		18	13.6
	Public green spaces maintenance		32	24.2

**Table 2:** Bivariate correlations and descriptive statistics of the variables.

	1	2	3	4	5	6	7	8	9	10	11	12	13	14	15	16	M	SD
(1) ACS	1																60.00	13.92
(2) ACS_1	0.828 **	1															12.80	3.32
(3) ACS_2	0.643 **	0.325 **	1														9.05	3.81
(4) ACS_3	0.762 **	0.495 **	0.482 **	1													12.52	3.73
(5) ACS_4	0.804 **	0.713 **	0.314 **	0.527 **	1												12.11	3.80
(6) ACS_5	0.749 **	0.643 **	0.275 **	0.408 **	0.565 **	1											13.74	3.50
(7) WRCS	0.483 **	0.400 **	0.149	0.339 **	0.403 **	0.574 **	1										93.75	19.12
(8) WRCS_A	0.457 **	0.394 **	0.093	0.390 **	0.432 **	0.447 **	0.891 **	1									50.09	9.24
(9) WRCS_A1	0.369 **	0.300 **	0.152	0.250 **	0.381 **	0.318 **	0.799 **	0.872 **	1								18.60	4.42
(10) WRCS_A2	0.403 **	0.329 **	0.105	0.431 **	0.307 **	0.346 **	0.730 **	0.850 **	0.599 **	1							16.11	3.09
(11) WRCS_A3	0.418 **	0.401 **	−0.037	0.359 **	0.423 **	0.513 **	0.735 **	0.830 **	0.538 **	0.638 **	1						15.38	3.32
(12) WRCS_B	0.424 **	0.328 **	0.164	0.232 **	0.311 **	0.606 **	0.927 **	0.652 **	0.609 **	0.500 **	0.527 **	1					44.00	11.07
(13) WRCS_B1	0.363 **	0.299 **	0.174	0.137	0.286 **	0.527 **	0.831 **	0.545 **	0.578 **	0.352 **	0.412 **	0.930 **	1				16.02	5.35
(14) WRCS_B2	0.381 **	0.293 **	0.096	0.270 **	0.232 **	0.587 **	0.810 **	0.544 **	0.433 **	0.472 **	0.491 **	0.895 **	0.767 **	1			14.45	3–34
(15) WRCS_B3	0.399 **	0.285 **	0.153	0.252 **	0.308 **	0.522 **	0.847 **	0.677 **	0.601 **	0.567 **	0.545 **	0.847 **	0.648 **	0.673 **	1		13.53	3.66
(16) IOS	0.503 **	0.413 **	0.274 **	0.373 **	0.498 **	0.377 **	0.515 **	0.548 **	0.469 **	0.501 **	0.435 **	0.380 **	0.346 **	0.287 **	0.381 **	1	25.84	4.65
(17) JSRS	0.075	0.117	−0.150	0.035	0.206 *	0.158	0.296 **	0.205 *	0.200 *	0.128	0.178	0.272 **	0.243 *	0.187	0.304 **	0.184	7.23	2.06

** Correlation is significant at the 0.01 level (2-tailed). * Correlation is significant at the 0.05 level (2-tailed). ACS = The Acceptance of Change Scale; ACS_1 = *Positive Reaction to Change*; ACS_2 = *Change Seeking*; ACS_3 = *Cognitive Flexibility*; ACS_4 = *Predisposition to Change*; ACS_5 = *Support for Change*; WRCS = Workplace Relational Civility Scale; WRCS_A = Workplace Relational Civility Scale part A; WRCS_A1 = *Relational Readiness* (part A); WRCS_A2 = *Relational Culture* (part A); WRCS_A3 = *Relational Decency* (part A); WRCS_B = Workplace Relational Civility Scale part A; WRCS_B1 = *Relational Readiness* (part B); WRCS_B2 = *Relational Culture* (part B); WRCS_B3: *Relational Decency* (part B); IOS = Insight Orientation Scale; JSRS: Job Satisfaction Rating Scale.

**Table 3:** Models effect indices.

Model	Total Effect	Direct Effect	Indirect Effect	Partial Standardized Indirect Effect	Completely Standardized Indirect Effect	Bootstrapping 95% CI	Sobel Test	
							z	*sign*
Model 1	0.10	0.05	0.04	0.02	0.09	(0.005, 0.106)	2.11	*p* < 0.05
Model 2	0.11	0.07	0.04	0.02	0.08	(–0.031, 0.117)	1.29	*p* = 0.20
Model 3	0.10	0.04	0.02	0.01	0.04	(0.001, 0.049)	-	
Model 4	0.10	0.04	0.00	0.00	0.00	(–0.010, 0.012)	-	

***Note***: Model 1 = the relationship between predisposition to change and job satisfaction, mediated by workplace relational civility part B; Model 2 = the relationship between predisposition to change and job satisfaction, mediated by insight orientation; Model 3 = the relationship between predisposition to change and job satisfaction, mediated by both insight orientation and workplace relational civility part B; Model 4: the relationship between predisposition to change and job satisfaction, mediated by both workplace relational civility part B and insight orientation.
